# Comprehensive bioinformatics-based annotation and functional characterization of bovine chymosin protein revealed novel biological insights

**DOI:** 10.1016/j.fochms.2023.100191

**Published:** 2023-12-27

**Authors:** Hafsa Amjad, Faiza Saleem, Munir Ahmad, Uzma Nisar, Hamza Arshad Dar

**Affiliations:** a Department of Biotechnology, Lahore College for Women University, Lahore 54590, Pakistan; b School of Biological Sciences, University of the Punjab, Quaid-e-Azam Campus, Lahore 54590, Pakistan; c Atta-ur-Rahman School of Applied Biosciences (ASAB), National University of Sciences and Technology (NUST), Sector H-12, Islamabad 44000, Pakistan

**Keywords:** Bovine chymosin, Cheese industry, Physiochemical analysis, Gene Ontology, Structural modeling, Functional characterization

## Abstract

Chymosin, an aspartic protease present in the stomachs of young ruminants like cows (bovine), causes milk coagulation and cheese production through the breakdown of κ-casein peptide bonds at the Met105-Phe106 site. Bovine chymosin is first synthesized as a pre-prochymosin that is cleaved to produce the mature chymosin protein. Despite significant strides in research, our understanding of this crucial enzyme remains incomplete. The purpose of this work was to perform *in silico* evolutionary and functional analysis and to gain unique insights into the structure of this protein. For this, the sequence of *Bos taurus* chymosin from UniProt database was subjected to various bioinformatics analyses. We found that bovine chymosin is a low molecular weight and hydrophilic protein that has homologs in other Bovidae species. Two active sites of aspartic peptidases, along with a functional domain, were identified. Gene Ontology analysis further confirmed chymosin's involvement in proteolysis and aspartic endopeptidase activity. Potential disordered residues and post-translational modification sites were also uncovered. It was revealed that the secondary structure of bovine chymosin is comprised of beta strands (44.27%), coils (43.65%), and alpha helices (12.07%). A highly optimized 3D structure was also obtained. Moreover, crucial protein–protein interactions were unveiled. Altogether, these findings provide valuable insights that could guide future research on bovine chymosin and its biological roles.

## Highlights

• Bovine chymosin is overall conserved.
• We identified two peptidase active sites and a functional domain.
• Secondary structure has many **β-**strands (44.27%) and coils (43.65%).

## 1 Introduction

Chymosin, a complex aspartic protease found in mammalian milk, plays a crucial role in cheese production by creating milk clots (Claverie-MartÌn & Vega-Hernàndez, 2007). Therefore, it is widely used in dairy industries for cheese preparation. This enzyme has a hydrolytic effect on peptide bonds among the amino acids Phe106-Met105 of κ-casein (Reid et al., 1997). Chymosin has been identified in several species of mammals such as pigs, camel, equine, rat, seal, and New World monkeys (Uniacke-Lowe & Fox, 2017a).

Initially, this enzyme is secreted as bovine pre-prochymosin, a larger protein containing 381 amino acids (Dekker, 2019). The first 16 amino acids form a hydrophobic signal peptide, separated upon secretion into the stomach, leaving behind prochymosin with 365 amino acids. This protein automatically activates upon exposure to gastric pH, losing an activation peptide from its amino end and converting it into a fully functional chymosin.

Bovine chymosin has been extensively studied by researchers (Langholm Jensen et al., 2013, Palmer et al., 2010). Although its role in milk clotting and cheese production is well-known, our understanding of its physiochemical characterization, evolutionary background, protein-protein interactions, and biological functions remains limited (Palmer et al., 2010, Uniacke-Lowe and Fox, 2017b). Moreover, there is limited information available on which sequence regions or individual amino acids are key functional residues of the protein.

Bioinformatics can be used to forecast the structural and functional characteristics of proteins. Numerous studies explored bioinformatics approaches to understand protein sequence conservation, evolutionary relationships, functional domains, active sites, physiochemical properties, and so on (Lagunas-Rangel, 2023, Malik et al., 2020, Narang et al., 2021). After examining the relevant literature, we could not find adequate information concerning these crucial aspects of bovine chymosin. Therefore, we undertook this study to perform a comprehensive bioinformatics-based annotation and characterization of bovine chymosin, including the generation of a high-quality 3D structure for understanding sequence-structure-function relationships. This not only provides valuable insights into bovine chymosin but also offers a general framework for investigating protein functions within their biological contexts. We believe our work serves as a valuable addition to previous research and opens doors for experimentalists to delve deeper into specific areas of bovine chymosin's function and potential applications, advancing our understanding of this critical enzyme.

## 2 Materials and methods

### 2.1 Protein sequence retrieval and phylogenetic analysis

The amino acid sequence of bovine chymosin (ID P00794, CHYM_BOVIN) was obtained from the UniProt database (Consortium, 2019). An NCBI BLAST search using default parameters was conducted on the mature protein sequence, excluding bovine sources. The top 60 non-bovine hits were downloaded and aligned with the query sequence using MUSCLE (Edgar, 2004) within MEGA11 (Tamura et al., 2021). Phylogenetic analysis was performed using the Neighbor-joining method with 1000 bootstrap iterations. The resulting tree was edited using the iTOL server (Letunic & Bork, 2021), a tool frequently employed for enhanced visualization of phylogenies (Ullah et al., 2019, Ullah et al., 2022, Zaheer et al., 2020).

### 2.2 Physicochemical characterization

The ExPasy tool ProtParam was used to assess various physical and chemical parameters associated with bovine chymosin (Gasteiger et al., 2003). The parameters determined included molecular weight, isoelectric value, GRAVY index, instability index, and aliphatic index. The GRAVY value provides information about protein hydrophilicity (Jaspard & Hunault, 2014), while the instability index indicates protein stability and the aliphatic index offers insights into potential thermal characteristics (Aziz et al., 2023).

### 2.3 Functional annotation of protein

To uncover potential functions, we employed the InterPROScan server (Jones et al., 2014). Annotations for subcellular location, biological process, and molecular function were performed. InterPro yields functional annotation by categorizing proteins into families, identifying potential domains, and pinpointing crucial residues (Blum et al., 2021). InterPro reigns supreme as a go-to tool for protein family annotation, with widespread adoption across the global scientific community.

### 2.4 Prediction of disordered protein residues and post-translational modifications

To identify disordered internal protein regions, we utilized two tools: Dispro (Cheng et al., 2005) and DISOPRED (Ward et al., 2004), employing default settings for analysis. To uncover potential N-glycosylation sites, we uploaded the amino acid sequence of chymosin to the NetNGlyc server (Gupta & Brunak, 2001). This server utilizes neural networks to explore Asn-Xaa-Ser/Thr sequons. Additionally, we employed the NetPhos 3.1 server (Blom et al., 1999) to predict potential phosphorylation sites in the enzyme, focusing on probable serine, threonine, or tyrosine residues. The threshold was set at 0.5.

### 2.5 Structural characterization and interaction analysis

To predict the secondary structure, we submitted the FASTA-formatted file to the PSIPRED 4.0 server using default settings (Buchan and Jones, 2019). This widely used service is estimated to process around 250,000 jobs annually for users worldwide (Buchan and Jones, 2019). To identify potential three-dimensional structures, we submitted the sequence to the NCBI PSI-BLAST service, searching against the Protein DataBank (Burley et al., 2018). A suitable structure with a PDB ID was then selected. To refine the quality of the 3D structure, we employed the GalaxyRefine server (Heo et al., 2013), a robust tool with citations in over 800 research papers. Structural quality parameters were assessed using PROCHECK (Laskowski et al., 1993), ERRAT (Colovos & Yeates, 1993), and ProSA web (Wiederstein & Sippl, 2007), consistent with verification procedures employed in other structural modeling studies (Dar et al., 2020, Shafaghi et al., 2023). The finalized protein structure was visualized using the standalone tool UCSF Chimera (Pettersen et al., 2004).

To predict potential interacting partners of chymosin, the STRING server was used. STRING is the most popular open-source protein–protein interaction network database that contains data for more than 10,000 different genomes (Szklarczyk et al., 2023). *Bos taurus* (bovine) was selected as the query organism and default parameters were chosen. Protein-protein interactions are fundamental to biological processes and can offer valuable insights into the functional roles of proteins (Naz et al., 2015).

## 3 Results

### 3.1 Evolutionary relationships of chymosin

**Fig. 1:**
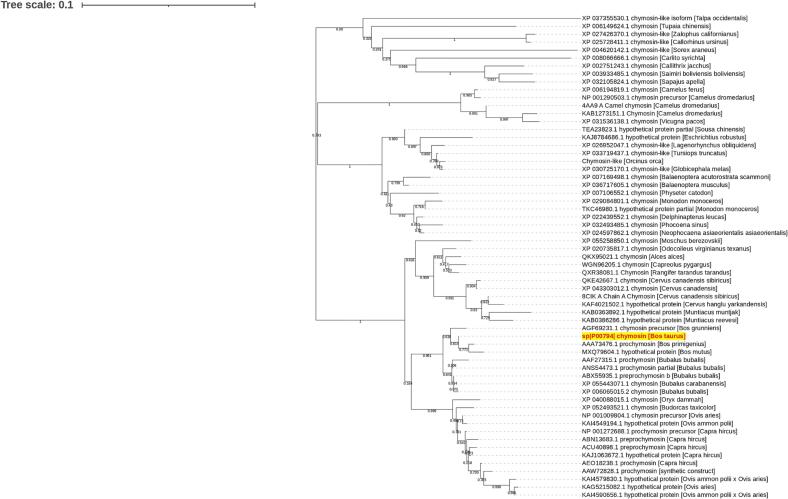
The evolutionary history was inferred as per the Neighbor-Joining method. The optimal tree is visualized. The query sequence is shown in red color with a yellow background for easy visualization. The tree is drawn to scale, with branch lengths in the same units as those of the evolutionary distances used to infer the phylogenetic tree. The evolutionary distances were computed by the Poisson correction method and are in the units of the number of amino acid substitutions per site. This analysis was conducted on 61 amino acid sequences. All ambiguous positions were removed for each sequence pair (pairwise deletion option). There was a total of 561 positions in the final dataset. (For interpretation of the references to color in this figure legend, the reader is referred to the web version of this article.)

Multiple sequence alignment revealed that bovine chymosin is highly conserved, with homologs present in other Bovidae species. Phylogenetic analysis of chymosin (Fig. 1) further confirmed this, highlighting a distinct clade dominated by Bovidae family members *Bos primigenius*, *Bos grunniens*, and *Bos mutus*. This clade was distinct from other main clades that contained proteins from other organisms. The evolutionary history was inferred by using the Neighbor-Joining method (Saitou & Nei, 1987) and the Poisson model (Zuckerkandl & Pauling, 1965). The tree with the highest likelihood was visualized (Fig. 1). The percentage of replicate trees in which the associated taxa clustered together in the bootstrap test (1000 replicates) are shown next to the branches (Felsenstein, 1985).

### 3.2 Physicochemical analysis

Physicochemical analysis revealed that chymosin enzyme has a molecular weight of 35652.92 Daltons whereas it has 4.42 computed isoelectric point. Therefore, it is anticipated to have slightly acidic behavior. Due to the presence of methionine at the N-terminal end of the sequence, the half-life was predicted to be 30 h in case of mammalian reticulocytes (in vitro), more than 20 h in case of yeast (in vivo), and more than 10 h in case of *Escherichia coli* (in vivo). The instability index was found to be 39.85, indicating the stable nature of enzyme. Moreover, an aliphatic index of 78.70 was revealed which projects reasonable thermostability of protein. The Grand average of hydropathicity (GRAVY) index was found to be −0.115, thus it is anticipated that the enzyme is predominantly hydrophilic and can interact favorably with water molecules (Dar et al., 2019).

### 3.3 Functional annotation

INTERPROScan predicted a singular biological process for the protein: proteolysis (GO:0006508). Additionally, it assigned the molecular function of aspartic-type endopeptidase activity (GO:0004190). No cellular component prediction was made. Structurally, residues 6–321 constitute a pepsin catalytic domain, while residues 31–42 and 213–224 form the aspartic peptidase active sites. Residues 3–322 exhibit homology to the aspartic peptidase domain superfamily of proteins.

### 3.4 Prediction of disordered protein residues and post-translational modifications

Dispro predicted disorder in the first two residues, glycine and glutamic acid, with probability values of 0.75 and 0.53, respectively. However, DISOPRED did not identify any disordered residues exceeding the threshold of 0.5.

**Fig. 2:**
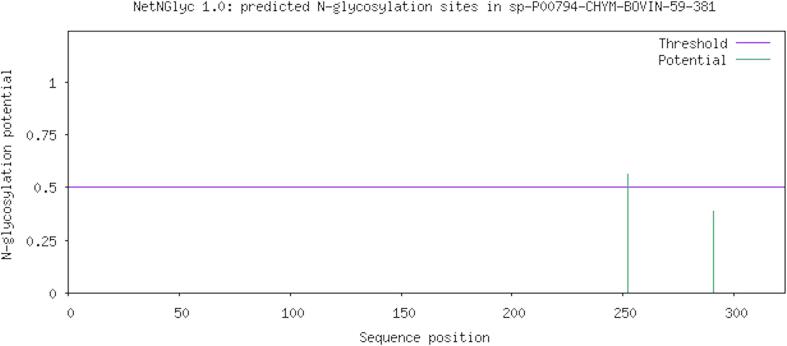
Predicted N-glycosylation sites in bovine chymosin.

**Fig. 3:**
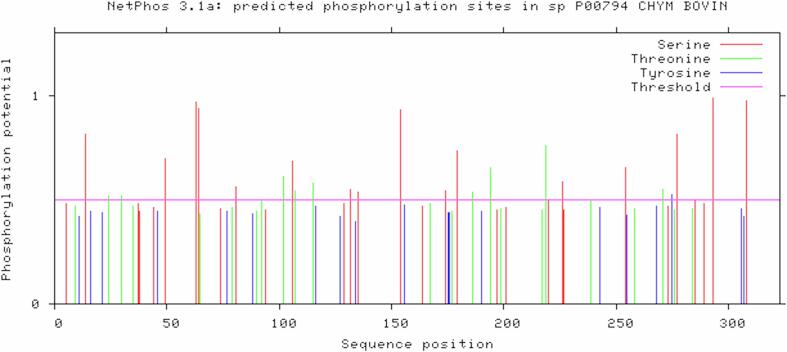
Predicted phosphorylation sites at Serine, Threonine, and Tyrosine positions in bovine chymosin.

The sequence of chymosin was analyzed to uncover potential N-glycosylation sites. Asn-Xaa-Ser/Thr sequons and predicted N-glycosylated Asparagines were highlighted. The NLSY sequon at position 252 had a score of 0.5606 (above 0.5 threshold) while the NHSQ sequon at position 291 scored 0.3878 (below 0.5 threshold). These findings are visually depicted in Fig. 2. NetPhos predicted a total of 43 kinase-specific putative phosphorylation sites (Supplementary File 1), as visualized in Fig. 3.

### 3.5 Structural characterization

**Fig. 4:**
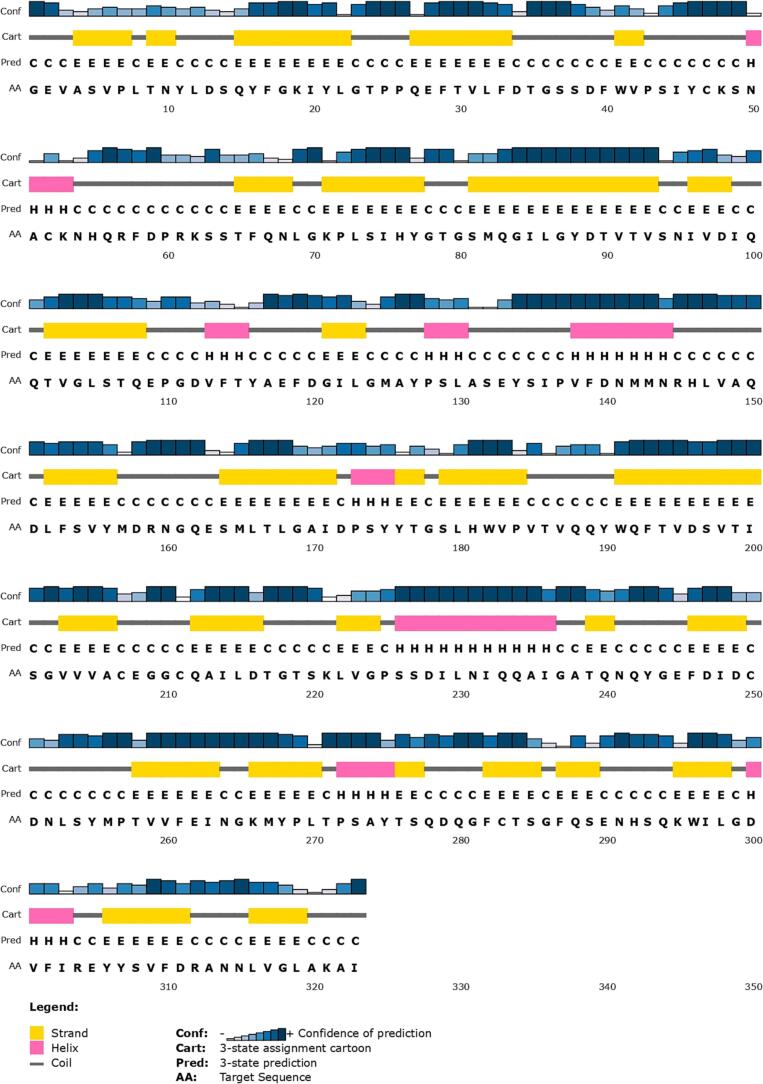
Figure visualizing secondary structure elements such as alpha helices, beta strands, and coils within the chymosin enzyme along with their confidence of prediction.

Secondary structural analysis of chymosin (Fig. 4) revealed its structural composition and organization, dominated by beta strands (∼44.27 %) and coils (∼43.65 %), with a few alpha helices (12.07 %).

**Fig. 5:**
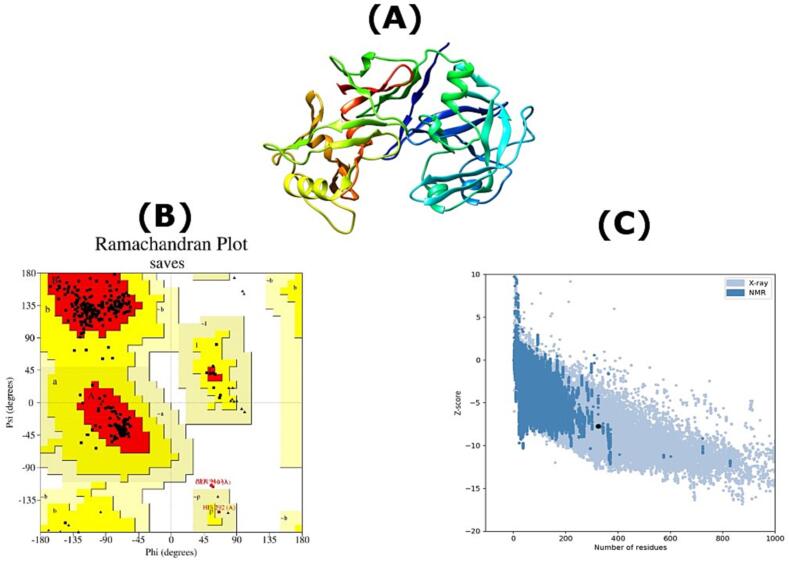
(A) Three-dimensional structure of bovine chymosin colored in rainbow style representation. (B) The Ramachandran plot of structure by the PROCHECK. (C) ProSA-based analysis and Z-score of −7.73.

Upon examining PSI-BLAST results, a suitable 3D crystal structure with PDB ID 1CMS was identified. This structure had an ERRAT quality score of 82.4841, indicating 86% and 12.5% residues in its core and allowed regions, respectively. We further refined the structural quality using molecular refinements, significantly improving it (Fig. 5A). The final model had an ERRAT score of 92.567, with 93.2% and 5.7% residues in the core and allowed regions, respectively (Fig. 5B). Notably, it also exhibited an overall near-native structure, closely resembling similar-sized NMR structures in the PDB database (Fig. 5C).

### 3.6 Protein-Protein interactions

**Fig. 6:**
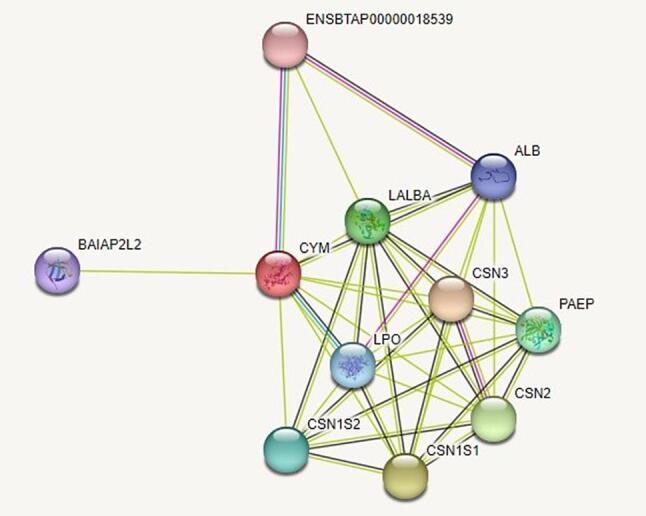
Protein-protein interaction analysis of bovine chymosin enzyme (red) with proteins from the STRING database. (For interpretation of the references to color in this figure legend, the reader is referred to the web version of this article.)

Protein-protein interactions are vital for many cellular processes inside living organisms. A search of the STRING database revealed ten functional interacting partners for the query protein bovine chymosin (Fig. 6). Notably, most of these interacting proteins, like chymosin itself, appear to have functions directly related to milk processing. This finding reaffirms a potential role for chymosin in a coordinated network of milk-processing enzymes.

## 4 Discussion

Understanding the functions of bovine chymosin enzyme is necessary to get updated information about its biological role and the overall significance in the life of *Bos taurus* (domestic cattle). Numerous studies have focused on the milk coagulation properties of this enzyme and elaborated on its utility in the cheese industry (Beltrán-Espinoza, 2021, Kappeler et al., 2006, Mbye et al., 2020). However, a detailed characterization of chymosin was found to be lacking in the literature. Therefore, we conducted a comprehensive bioinformatic analysis of this crucial protein using a range of bioinformatics tools.

Recombinant technology has been used by researchers to engineer novel chymosin enzymes with tailored properties for specific applications (Akishev et al., 2022, Vallejo et al., 2012, Wei et al., 2016). Notably, Vallejo et al. demonstrated that recombinant goat chymosin displayed superior catalytic efficiency compared to buffalo, bovine, or camel versions (Vallejo et al., 2012). In another study, Grahame et al. used bioinformatic tools to compare the structure and sequence of renin and pepsin (Grahame et al., 2020). By analyzing key residues, motifs, solvent exposure, and flexibility, they attempted to understand why aspartic proteases are stable at different pH levels. This trend highlights the potential of tailoring chymosin enzymes for specific applications, which further underscores the need for comprehensive annotation and characterization of the enzyme.

Multiple sequence alignment confirmed that chymosin is relatively conserved and has homologs in other Bovidae species as well. Phylogenetic analysis revealed the evolutionary relationships between bovine chymosin and its homologs, with all Bos subgenera chymosins forming a distinct sub-clade within the phylogenetic tree. Additionally, evolutionary similarities were observed between bovine chymosin and chymosin proteins from *Bubalus bubalis* and *Bubalus carabanensis*.

Physiochemical analysis projected this to be a low-molecular-weight protein (around 35 kDa) with acidic properties (pI below 4.5). The presence of methionine at the N-terminal end suggests a potentially long half-life, exceeding 30 h. Additionally, the protein is predicted to be predominantly hydrophilic and exhibit thermostability, indicating potential robustness against temperature changes.

After applying molecular simulations, the refined bovine chymosin structure exhibited significantly enhanced quality, with an ERRAT score exceeding 92 compared to the initial 82. Ideally, at least 90% of residues in a suitable protein structure should fall within the most favored regions of the Ramachandran plot (Rai & Rieder, 2012), and with over 93% residues, our selected model exceeded this benchmark. The near-native configuration was further validated by the ProSA-web graph analysis. Moreover, crucial protein-protein interactions were unveiled.

## 5 Conclusions

In this study, we conducted a comprehensive *in silico* characterization of bovine chymosin. Due to its role in milk coagulation and cheese production, it is essential for the dairy industry. We found that bovine chymosin is a stable, hydrophilic, and low molecular weight protein that has homologs in other Bovidae species. Further analysis revealed the presence of a catalytic domain and two active sites as well as the involvement of enzyme in proteolysis and aspartic endopeptidase activity. Crucial internal disordered residues and post-translational modification sites were also uncovered. As per secondary structure analysis, beta strands and coils are frequently found in protein. Through molecular simulations, a highly optimized 3D structure was also obtained and verified. Finally, important interacting partners of bovine chymosin were identified. The present study was helpful to enhance our understanding of bovine chymosin, an important yet poorly understood protein. While computational approaches provide a solid theoretical foundation, ideally they should be accompanied by wet laboratory validation experiments. More follow-up studies are needed to update the researchers about these matters.

## CRediT authorship contribution statement

**Hafsa Amjad:** Conceptualization, Investigation, Writing – original draft, Writing – review & editing. **Faiza Saleem:** Investigation, Supervision, Writing – original draft, Writing – review & editing. **Munir Ahmad:** Investigation, Methodology, Resources. **Uzma Nisar:** Software, Writing – review & editing. **Hamza Arshad Dar:** Methodology, Writing – review & editing.

## Declaration of competing interest

The authors declare that they have no known competing financial interests or personal relationships that could have appeared to influence the work reported in this paper.

## Data Availability

All the data has been included in the manuscript and supplementary files.
