# Supplementary material for: Comprehensive bioinformatics-based annotation and functional characterization of bovine chymosin protein revealed novel biological insights
Source: Food Chem (Oxf). 2023 Dec 27;8:100191. doi: 10.1016/j.fochms.2023.100191 (PMC10801198; doi:10.1016/j.fochms.2023.100191)
Supplement: Supplementary data 1 [file mmc1.docx]

**Supplementary material**

**Supplementary Table 1.** Table showing kinase-specific phosphorylation sites in bovine chymosin along with their respective scores.

| **Position** | **Residue** | **Context** | **Score** | **Kinase** |
| --- | --- | --- | --- | --- |
| 14 | S | NYLDSQYFG | 0.816 | unspecified |
| 14 | S | NYLDSQYFG | 0.556 | DNAPK |
| 24 | T | IYLGTPPQE | 0.518 | p38MAPK |
| 30 | T | PQEFTVLFD | 0.52 | cdc2 |
| 49 | S | IYCKSNACK | 0.693 | PKC |
| 63 | S | DPRKSSTFQ | 0.968 | unspecified |
| 63 | S | DPRKSSTFQ | 0.559 | PKA |
| 63 | S | DPRKSSTFQ | 0.528 | RSK |
| 64 | S | PRKSSTFQN | 0.938 | unspecified |
| 64 | S | PRKSSTFQN | 0.802 | PKA |
| 64 | S | PRKSSTFQN | 0.674 | PKC |
| 81 | S | YGTGSMQGI | 0.56 | DNAPK |
| 102 | T | DIQQTVGLS | 0.61 | PKC |
| 106 | S | TVGLSTQEP | 0.685 | unspecified |
| 107 | T | VGLSTQEPG | 0.539 | DNAPK |
| 115 | T | GDVFTYAEF | 0.58 | CKII |
| 115 | T | GDVFTYAEF | 0.572 | PKG |
| 132 | S | PSLASEYSI | 0.546 | unspecified |
| 135 | S | ASEYSIPVF | 0.537 | CKI |
| 154 | S | QDLFSVYMD | 0.928 | unspecified |
| 154 | S | QDLFSVYMD | 0.519 | PKC |
| 174 | S | AIDPSYYTG | 0.541 | DNAPK |
| 174 | S | AIDPSYYTG | 0.519 | cdc2 |
| 179 | S | YYTGSLHWV | 0.736 | PKC |
| 186 | T | WVPVTVQQY | 0.536 | PKC |
| 194 | T | YWQFTVDSV | 0.656 | unspecified |
| 194 | T | YWQFTVDSV | 0.515 | PKC |
| 219 | T | LDTGTSKLV | 0.759 | PKC |
| 219 | T | LDTGTSKLV | 0.644 | unspecified |
| 220 | S | DTGTSKLVG | 0.502 | cdc2 |
| 226 | S | LVGPSSDIL | 0.587 | PKA |
| 239 | T | AIGATQNQY | 0.502 | DNAPK |
| 254 | S | CDNLSYMPT | 0.654 | unspecified |
| 271 | T | MYPLTPSAY | 0.546 | p38MAPK |
| 271 | T | MYPLTPSAY | 0.501 | GSK3 |
| 275 | Y | TPSAYTSQD | 0.523 | EGFR |
| 277 | S | SAYTSQDQG | 0.815 | unspecified |
| 277 | S | SAYTSQDQG | 0.587 | ATM |
| 277 | S | SAYTSQDQG | 0.582 | DNAPK |
| 293 | S | SENHSQKWI | 0.986 | unspecified |
| 293 | S | SENHSQKWI | 0.624 | DNAPK |
| 293 | S | SENHSQKWI | 0.609 | ATM |
| 308 | S | REYYSVFDR | 0.972 | unspecified |
